# Prediction Model for eGFR Thresholds Guiding the Optimal Timing of Hemodialysis Preparation in Chronic Kidney Disease

**DOI:** 10.3390/biomedicines13122960

**Published:** 2025-12-01

**Authors:** Geo Neul Park, Yoonwon Choi, Ji Eun Moon, Seon Min Kim, Jin Kuk Kim, Moo Yong Park, Soo Jeong Choi, Byung Chul Yu

**Affiliations:** 1 Division of Nephrology, Department of Internal Medicine, Soonchunhyang University Bucheon Hospital, Soonchunhyang University College of Medicine, 170, Jomaru-ro, Bucheon 14584, Republic of Korea; geoneul@schmc.ac.kr (G.N.P.); 124940@schmc.ac.kr (Y.C.); 121746@schmc.ac.kr (S.M.K.); medkjk@schmc.ac.kr (J.K.K.); mypark@schmc.ac.kr (M.Y.P.); crystal@schmc.ac.kr (S.J.C.); 2 Department of Biostatistics, Clinical Trial Center, Soonchunhyang University Bucheon Hospital, 170, Jomaru-ro, Bucheon 14584, Republic of Korea; moon6188@schmc.ac.kr

**Keywords:** chronic kidney disease, end-stage kidney disease, hemodialysis, kidney replacement therapy preparation, prediction model

## Abstract

**Background/Objectives**: The progression of chronic kidney disease (CKD) is influenced by multiple factors, complicating the determination of the optimal timing for hemodialysis preparation. The aim of this study was to identify predictive factors and develop a model to guide this timing in patients with CKD. **Methods**: This retrospective study included patients who progressed to end-stage kidney disease (ESKD) and initiated hemodialysis after at least one year of follow-up at a single tertiary hospital between January 2011 and June 2024. The estimated glomerular filtration rate at 6 months before hemodialysis initiation (eGFR_6M), indicating timing for vascular access creation, and its decline trajectory were retrospectively analyzed according to underlying diseases and clinical conditions. A regression model was developed, and its performance was evaluated in internal and external validation cohorts. **Results**: Among 507 patients, the mean eGFR_6M was 11.7 ± 4.9 mL/min/1.73 m^2^, with higher values observed in patients with diabetes mellitus (DM), cardiovascular disease (CVD), stroke, dementia, liver cirrhosis (LC), nephrotic-range proteinuria, or hypoalbuminemia. The mean eGFR_6M decline rate was 8.3 ± 9.6 mL/min/1.73 m^2^/year, with more rapid declines observed in patients with DM, LC, nephrotic range proteinuria, and hypoalbuminemia. The model was developed using significant predictors—sex, impaired mobility, DM, CVD, left ventricular ejection fraction, blood urea nitrogen, and phosphate levels—and showed acceptable performance in both validation cohorts, with P30 ranging from 70% to 75%. **Conclusions**: This study provides nephrologists with an objective reference to guide the timing of dialysis preparation, supporting personalized ESKD life planning and improving patient outcomes.

## 1. Introduction

The prevalence of end-stage kidney disease (ESKD) continues to rise annually, with 19,286 newly diagnosed cases reported in South Korea and 135,972 in the United States in 2021 [1,2]. According to 2023 data from South Korea, 89% of patients with ESKD received dialysis, with 84% undergoing hemodialysis and 5% undergoing peritoneal dialysis [2]. Despite the widespread use and clinical significance of hemodialysis, determining the optimal timing for vascular access creation in patients with chronic kidney disease (CKD) remains a major challenge. As the progression to ESKD is influenced by various factors, dialysis initiation should be planned with consideration not only for kidney function but also for comorbidities and functional status [3,4,5,6,7].

Although numerous studies have been conducted to guide the timing of dialysis preparation, unplanned hemodialysis via central venous catheter remains the most common initial modality, accounting for 55.7% of cases [8]. This unprepared approach is associated with significantly higher morbidity and mortality compared with planned dialysis initiation using appropriate vascular access [9,10,11,12,13]. On the other hand, premature vascular access creation may lead to unnecessary surgical procedures and access-related complications, further complicating decisions about when to initiate dialysis preparation [14,15,16].

Existing guidelines on the timing of dialysis preparation remain inconsistent. The 2006 Kidney Disease Outcomes Quality Initiative (KDOQI) guidelines recommended early planning in patients with CKD stage 4, defined as estimated glomerular filtration rate (eGFR) < 30 mL/min/1.73 m^2^ [17]. In the same year, the Canadian Society of Nephrology guidelines recommended vascular access creation at a creatinine clearance of 15–20 mL/min [18]. Although no prospective studies have established optimal eGFR thresholds for dialysis preparation, one well-conducted simulation study proposed that an eGFR range of 15–20 mL/min/1.73 m^2^ may be appropriate [19]. Meanwhile, the updated 2019 KDOQI guidelines no longer specify eGFR cutoffs. Instead, they advocate initiating preparation 6 to 9 months before the anticipated need, based on patient age, comorbidities, and functional status [20].

Despite this shift toward personalized care, practical and objective guidance remains limited. Consequently, decisions regarding the timing of dialysis preparation often rely on the experience of individual nephrologists. Developing an evidence-based framework that integrates eGFR and patient-specific factors could lead to more timely and effective initiation of dialysis. To address this need, we aimed to explore influential factors for dialysis preparation timing and develop a predictive model in patients with CKD, thereby contributing to improved clinical decision-making and patient outcomes.

## 2. Materials and Methods

### 2.1. Study Population

**Figure 1:**
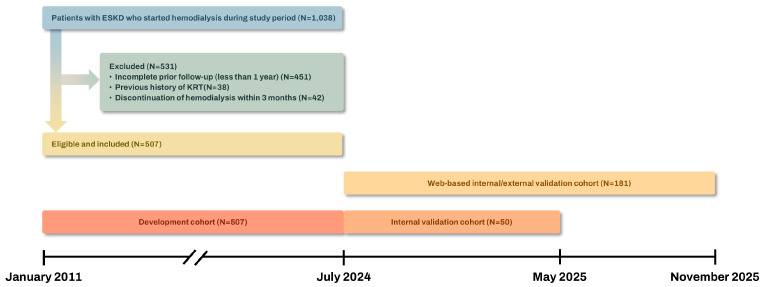
Study population. ESKD, end-stage kidney disease; KRT, kidney replacement therapy.

This retrospective observational study was conducted among a cohort of patients diagnosed with ESKD who initiated hemodialysis between January 2011 and June 2024 at Soonchunhyang University Bucheon Hospital. ESKD was defined as the initiation of maintenance hemodialysis as decided by the attending nephrologist, indicating the need for chronic kidney replacement therapy (KRT). Patients who were not followed for at least one year for CKD in the nephrology clinic or had a prior history of KRT were excluded. Patients who discontinued hemodialysis within three months of initiation—owing to transition to other KRTs (such as peritoneal dialysis or kidney transplantation), death, or recovery of kidney function—were also excluded. A total of 507 patients were finally included in the study (Figure 1).

Model validation was conducted in two stages. An initial validation was conducted using an independent internal cohort, followed by web-based internal and external validation. An independent internal validation cohort consisted of 50 patients diagnosed with ESKD who initiated hemodialysis between July 2024 and April 2025 at Soonchunhyang University Bucheon Hospital. To facilitate clinical application and further evaluate the generalizability of the model, we implemented the final prediction equation as a simple web-based calculator (https://v0-e-gfr-calculator.vercel.app/ (accessed on 21 October 2025)) (Supplementary Figure S1) and applied it at three tertiary care hospitals within the Soonchunhyang University Medical Center (Soonchunhyang University Bucheon, Cheonan, and Seoul Hospitals). In this setting, Bucheon served as the site for a second internal validation, while Cheonan and Seoul constituted the external validation cohort. We included incident HD patients who initiated HD between July 2024 and November 2025 at the external validation hospitals and between May 2025 and November 2025 at Bucheon. In total, an additional 181 patients were evaluated across these three centers (Figure 1). The same inclusion and exclusion criteria were applied to all validation cohorts as to the development cohort.

This study was conducted according to the principles expressed in the Declaration of Helsinki. Clinical data were obtained from electronic medical records with the approval of the Institutional Review Board (IRB) of Soonchunhyang University Bucheon Hospital (IRB no. 2025-04-007-001). Written consent was waived by the IRB because of the retrospective nature of the study, and all data were fully anonymized before access by the researchers.

### 2.2. Clinical and Laboratory Data

Data on demographics, anthropometric measurements, physical function, comorbidities, medications, echocardiographic findings, and chest X-ray findings were collected 1 year prior to hemodialysis initiation. Laboratory data were collected at the following time points: annually from 5 months to 1 year prior to hemodialysis initiation; at 6 months, 3 months, and 1 month prior; and at the time of initiation. Furthermore, eGFR was calculated using the Chronic Kidney Disease Epidemiology Collaboration equation [21], and the mean annual rate of eGFR decline was calculated as the average of the difference in eGFR levels between each year. Proteinuria was assessed using the protein-creatinine ratio in random urine samples, and nephrotic-range proteinuria was defined as a protein-creatinine ratio exceeding 3.5 g/g. Hypoalbuminemia was defined as a serum albumin level < 3.3 g/dL, and volume overload status was defined as a condition requiring diuretic therapy. Poorly controlled diabetes mellitus (DM) was defined as a glycated hemoglobin level exceeding 7% in patients younger than 65 years and exceeding 7.5% in patients aged 65 years or older. Physical activity was classified into three categories: independent mobility, requiring partial assistance, and requiring absolute assistance. The stage of requiring absolute assistance was defined as impaired mobility.

### 2.3. Key Variables of Interest and Prediction Model Development

Following the 2019 KDOQI guidelines, eGFR assessed 6 months before hemodialysis initiation (eGFR_6M) was defined as the appropriate time point for vascular access creation; therefore, it was selected as the primary variable of interest in this study [20]. The mean eGFR_6M and trajectory of eGFR decline were analyzed according to the comorbidities, administered medications, and clinical conditions of the patients. These analyses enabled the identification of differences in the patterns of eGFR change associated with specific clinical conditions.

In addition, a prediction model was developed to estimate eGFR_6M based on demographics, physical abilities, comorbidities, echocardiographic findings, and laboratory data of the patients, and was validated using internal and external cohorts.

### 2.4. Statistical Analysis

Continuous variables with a normal distribution were expressed as mean ± standard deviation and compared using the independent *t*-test. Categorical variables were presented as frequencies and percentages and analyzed with Pearson’s chi-square test or Fisher’s exact test, as appropriate. To evaluate potential temporal changes in hemodialysis initiation practices, we compared mean eGFR_6M and eGFR at the time of hemodialysis initiation (eGFR_0) across calendar periods using one-way analysis of variance (ANOVA). Furthermore, the patterns of eGFR decline before hemodialysis initiation, stratified by patient factors, were analyzed using a linear mixed model.

To develop the prediction model of eGFR_6M, univariate and multivariate linear regression models were utilized. Significant variables from the univariate analysis were included in the multiple linear regression model. Model performance was assessed using the percentage of predicted values within 30% of the observed values (P30), the mean absolute error (MAE), and the root mean squared error (RMSE).

A *p*-value less than 0.05 was considered statistically significant. All analyses were performed using SPSS 27 (SPSS Inc., Chicago, IL, USA), GraphPad Prism 10 (GraphPad Inc., La Jolla, CA, USA), and Rex 3.6.3 (RexSoft Inc., Seoul, Republic of Korea). A post hoc power analysis for multiple linear regression (fixed model, R^2^ deviation from zero) was conducted based on Cohen’s f^2^. Using 7 predictors (u = 7), residual degrees of freedom of 499 (v = 499), an effect size of f^2^ = 0.56 (corresponding to R^2^ ≈ 0.36), and a two-sided significance level of α = 0.05, the statistical power (1 − β) was calculated to be greater than 0.99, indicating that the sample size was sufficient for the primary regression analysis.

## 3. Results

### 3.1. Study Population

Table 1 presents the baseline characteristics of patients at the time of hemodialysis initiation. Among the total of 507 patients, males were predominant, and the mean age was 61.0 ± 14.2 years. The mean duration from the first nephrologist visit to the initiation of hemodialysis was 5.7 ± 4.3 years. The mean proteinuria level at the time of hemodialysis initiation was 3.3 ± 2.7 g/g. Hypertension was present in most patients (95.7%), and more than half (58.4%) had DM. At the time of hemodialysis initiation, the mean eGFR was 6.2 ± 2.8 mL/min/1.73 m^2^.

### 3.2. Individualized eGFR Levels for Hemodialysis Preparation According to Patient Factors

The mean eGFR values before hemodialysis initiation in the overall patient population are summarized in Table 2. They were approximately 36 mL/min/1.73 m^2^ at 5 years before initiation, 28 mL/min/1.73 m^2^ at 3 years, 23 mL/min/1.73 m^2^ at 2 years, 16 mL/min/1.73 m^2^ at 1 year, and 12 mL/min/1.73 m^2^ at 6 months, ultimately reaching 6 mL/min/1.73 m^2^ at the start of dialysis.

In a time-stratified analysis according to the calendar period of hemodialysis initiation, neither mean eGFR_6M nor mean eGFR_0 differed significantly across years, and no clear temporal trend was observed (Supplementary Figure S2).

**Figure 2:**
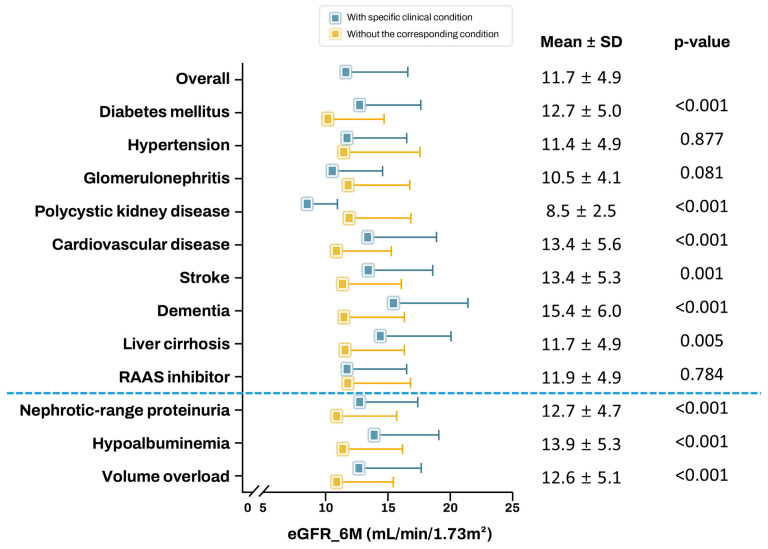
Mean estimated glomerular filtration rate 6 months before hemodialysis initiation (eGFR_6M) by clinical conditions of patients. Data are shown as mean ± standard deviation (SD). RAAS, renin–angiotensin–aldosterone system.

Mean eGFR_6M was analyzed according to comorbidities, administered medications, severity of proteinuria, and albumin levels (Figure 2). Patients with DM, cardiovascular disease (CVD), stroke, dementia, or liver cirrhosis (LC) required hemodialysis preparation at a higher eGFR_6M compared with those without these conditions. In contrast, patients with polycystic kidney disease (PCKD) required preparation at lower eGFRs. In addition, patients with nephrotic-range proteinuria, hypoalbuminemia, and volume overload required preparation at higher eGFRs. Patients with poorly controlled DM required hemodialysis preparation at an eGFR level 0.9 mL/min/1.73 m^2^ higher than patients with well-controlled DM (Supplementary Table S1). To exclude the confounding effect of DM on CVD, stroke, and proteinuria, a subgroup analysis was conducted on patients without DM. The mean eGFR_6M for these conditions remained higher in these patients (Supplementary Table S2).

### 3.3. Impact of Comorbidities and Clinical Conditions on the Trajectory of eGFR Decline

**Figure 3:**
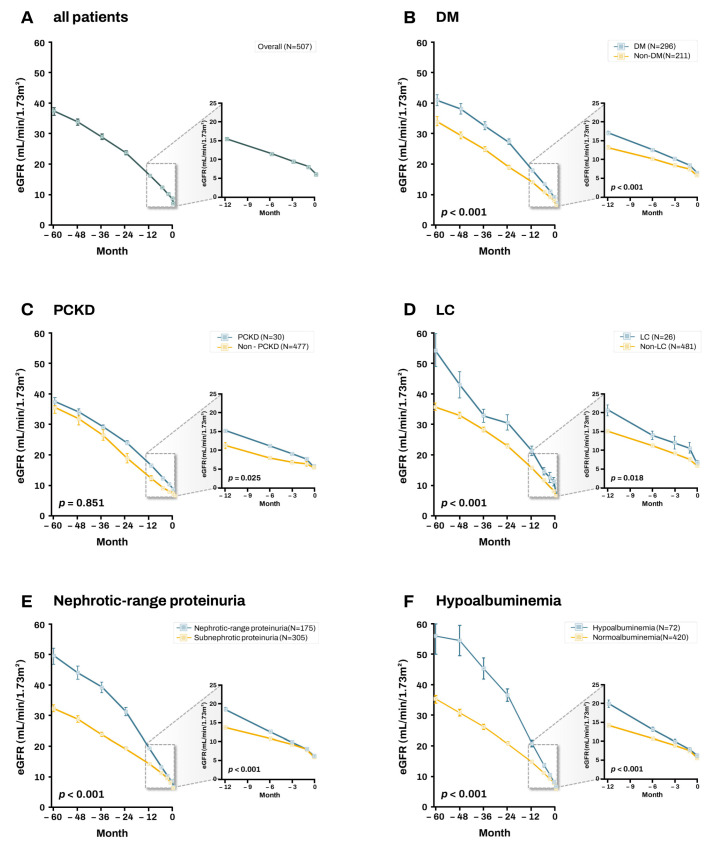
The trajectory of estimated glomerular filtration rate (eGFR) decline according to patient characteristics. Trajectories of eGFR decline in (**A**) all patients and according to the presence of (**B**) diabetes mellitus (DM), (**C**) polycystic kidney disease (PCKD), (**D**) liver cirrhosis (LC), (**E**) nephrotic-range proteinuria, and (**F**) hypoalbuminemia. Data are shown as mean ± standard error.

Figure 3 illustrates the trajectory of eGFR decline based on patient factors. On average, patients with a mean eGFR decline of 7.3 ± 7.9 mL/min/1.73 m^2^/year initiated hemodialysis within 1 year, whereas those with a mean decline of 8.3 ± 9.6 mL/min/1.73 m^2^/year initiated hemodialysis within 6 months. Patients with DM, LC, nephrotic-range proteinuria, and hypoalbuminemia exhibited a significantly faster eGFR decline compared with those without these conditions. These patients also showed a more rapid decline in eGFR compared with the overall cohort. Meanwhile, unlike other patient factors, PCKD was associated with a slower decline in eGFR compared with the non-PCKD group. In the subgroup analysis of patients with DM, poorly controlled DM was associated with a significantly greater eGFR decline compared with well-controlled DM (Supplementary Figure S3). To account for the confounding effect of DM, a subgroup analysis was performed on patients without DM, confirming that nephrotic-range proteinuria was independently associated with a more rapid decline in eGFR (Supplementary Figure S4).

### 3.4. Prediction Model for the Optimal Timing of Hemodialysis Preparation: Development and Validation

Univariate (Table 3) and multivariate (Table 4) linear regression analyses were performed using eGFR_6M as the dependent variable. Independent variables included demographics, anthropometric data, physical abilities, comorbidities, smoking status, echocardiographic findings, and laboratory variables. Based on significant variables, a prediction model was developed using data from 382 patients after excluding those with missing data.

The model demonstrated that dialysis preparation is required at higher eGFR levels in patients with any of the following individual characteristics: male sex, impaired mobility, DM, CVD, reduced ejection fraction (EF), or low levels of either blood urea nitrogen (BUN) or serum phosphorus (P).

eGFR_6M = 11.394 + 1.129 × (male) + 1.341 × (impaired mobility) +0.996 × (DM) + 1.047 × (CVD) − 0.068 × (EF) +3.315 × (if BUN ≤ 60) +2.717 × (if P ≤ 4.5)

The adjusted R^2^ of the model was 0.361, and the Durbin–Watson statistic was 2.131, indicating no autocorrelation. Scatter and quantile-quantile plots confirmed that the assumptions of normality, homoscedasticity, and linearity were met (Supplementary Figure S5).

In the internal validation cohort, all variables except CVD were comparable to those in the development cohort (Supplementary Table S3). When the predicted eGFR_6M derived from the regression equation was compared with the observed values, the model achieved a P30 of 70.0%, a MAE of 2.28 mL/min/1.73 m^2^, and a RMSE of 2.97 mL/min/1.73 m^2^. In addition, when the prediction equation was implemented as an online tool at three tertiary care hospitals, the P30, MAE, and RMSE were 75.4%, 2.37 mL/min/1.73 m^2^, and 3.14 mL/min/1.73 m^2^, respectively, which were comparable to the performance observed in the original internal validation cohort. In this web-based validation cohort, all variables except CVD and phosphorus level showed a similar distribution to those in the development cohort (Supplementary Table S4).

## 4. Discussion

The current study identified predictive factors for the timing of hemodialysis preparation in patients with CKD. At 6 months before hemodialysis initiation, the average eGFR was 11.7 ± 4.9 mL/min/1.73 m^2^, with a mean annual decline rate of 8.3 ± 9.6 mL/min/1.73 m^2^/year. Patients with DM, CVD, stroke, dementia, LC, nephrotic-range proteinuria, hypoalbuminemia, or volume overload required dialysis preparation at higher eGFRs. Similarly, patients with DM, LC, nephrotic-range proteinuria, or hypoalbuminemia exhibited a more rapid decline in eGFR. In contrast, patients with PCKD experienced a slower decline in eGFR, allowing dialysis preparation to be delayed until eGFR reached lower levels. In addition, a model was developed that incorporates sex, physical abilities, comorbidities, echocardiographic findings, and laboratory data, providing a predictive tool to determine the timing for hemodialysis preparation. The model was subsequently validated using internal and external validation cohorts. These findings offer valuable clinical guidance for nephrologists managing patients with CKD and optimizing hemodialysis preparation timing.

Given the substantial heterogeneity in CKD progression, the revised 2019 KDOQI guidelines emphasize an individualized approach to dialysis preparation, taking into account patient-specific factors such as age, comorbidities, and functional status [20]. In line with this approach, prior studies have attempted to model the risk of progression to ESKD using demographic and laboratory data [22,23,24,25]. However, these models were primarily focused on predicting the risk of disease progression rather than offering an objective framework to guide the timing of hemodialysis preparation. Additionally, they did not account for patient comorbidities. To address these limitations, we provided individualized reference eGFR values and developed a predictive model that integrates comorbidities, clinical conditions, laboratory data, and imaging findings.

Analysis of mean eGFR levels over time before hemodialysis initiation in the overall patient population revealed indicative timeframes. These generalized figures may serve as simple and intuitive reference points for explaining prognosis to patients with CKD at their initial outpatient visit, even in the absence of a confirmed etiology. Furthermore, they can offer nephrologists a practical guide to support clinical judgment when determining the timing of dialysis preparation. Patients with DM, CVD, stroke, dementia, LC, nephrotic-range proteinuria, hypoalbuminemia, or volume overload required dialysis preparation at higher eGFRs than average. These conditions align with previously established risk factors for CKD progression [3,4,5,6,7,26,27]. Notably, dementia—though not a conventional risk factor for CKD progression—was associated with the highest eGFR_6M, highlighting the potential influence of cognitive and functional status on disease progression. Meanwhile, in contrast to patients who delay dialysis initiation by their own decision, patients with dementia often begin dialysis based on decisions made by physicians and caregivers. Therefore, they tend to initiate dialysis at relatively higher eGFR levels without significant delay. Furthermore, hypoalbuminemia was associated with a higher eGFR_6M than nephrotic-range proteinuria, indicating that hypoalbuminemia may reflect malnutrition rather than simply being a consequence of proteinuria [28,29]. This underscores the importance of nutritional status in determining the timing of hemodialysis preparation. In contrast, patients with PCKD in the present study had lower eGFR_6M than the overall cohort. They had a slower disease progression than patients without PCKD, allowing them to maintain relatively stable clinical conditions and tolerate lower eGFRs without developing uremic symptoms [30].

Regarding the trajectory of eGFR decline, an annual eGFR decline rate of approximately ≥8 mL/min/1.73 m^2^/year was associated with dialysis initiation within 6 months. Additionally, patients with DM, LC, nephrotic-range proteinuria, and hypoalbuminemia had significantly faster eGFR decline rates compared with those without these conditions. These findings provide an intuitive reference for nephrologists in clinical practice when determining dialysis preparation timing.

The predictive model for eGFR_6M revealed that patients with male sex, impaired mobility, DM, CVD, reduced EF, and low levels of BUN or P were more likely to require earlier hemodialysis preparation. These findings align with those of the mean eGFR_6M analysis based on patient-specific factors. While nephrotic-range proteinuria and hypoalbuminemia showed close associations in this analysis, they were not included in the final prediction model owing to their low explanatory power. An unexpected finding from our model was that lower BUN and P levels were associated with earlier dialysis preparation. This can be explained by previous studies suggesting that low BUN and P levels in patients with advanced CKD may reflect underlying malnutrition [31,32,33]. The identification of impaired mobility as a significant variable in our model highlights the importance of functional status in CKD progression [34,35,36].

The adjusted R^2^ of the model was 0.361, which may be interpreted as modest. The P30 values of the two validation cohorts were 70.0% and 75.4%, which are somewhat lower than those reported for widely used eGFR estimating equations when validated against measured eGFR. However, since the model was designed to predict specific eGFR values rather than categorical risk, its explanatory power is inherently constrained. Given the biological complexity and inherent variability of eGFR, achieving precise point estimates is challenging. Therefore, in clinical practice, predictions that approximate the actual value are usually considered reliable by nephrologists. In this study, both the MAE and RMSE were within clinically acceptable ranges, further supporting the reliability of the model’s predictions. When the prediction equation was implemented as an online tool at three tertiary-care hospitals, the model demonstrated consistent accuracy, providing additional internal and external validation and supporting its generalizability and practical feasibility in real-world clinical practice.

This study has several limitations. First, as a retrospective observational study based on electronic medical records, it was subject to missing or incompletely recorded clinical data, and standardized assessments of symptoms or functional status at predefined time points were not available. Although we applied prespecified inclusion and exclusion criteria and limited our analyses to routinely collected variables, residual information bias and selection bias cannot be fully excluded. Second, both the development cohort and the internal validation cohort were derived from a single center, and the overall sample size was modest compared with large registry-based datasets. These factors inherently limit the generalizability of the prediction model. To partially address this limitation, we conducted additional validation using the web-based calculator across three tertiary-care hospitals. Nonetheless, larger and more diverse prospective multicenter studies are still warranted to further establish the robustness and generalizability of the model. Third, there are no universally accepted threshold values for key performance metrics used to evaluate eGFR prediction models—such as P30, MAE, and RMSE. In this study, model performance was interpreted in the context of previously published eGFR prediction work and in light of the intended role of the model as a supportive tool to assist, rather than replace, clinical decision-making. However, the absence of standardized benchmarks makes it difficult to define a clear cutoff for what should be regarded as “clinically acceptable” predictive accuracy.

Finally, evolving clinical practices and treatment patterns related to HD initiation over the 14-year study period may affect the applicability of the model to contemporary clinical settings. To address this concern, we performed a time-stratified analysis comparing eGFR_6M and eGFR_0 across calendar periods. Neither measure showed significant temporal variation, suggesting that the clinical threshold for initiating maintenance HD has remained relatively consistent over time and that the model is likely to remain applicable in current practice.

## 5. Conclusions

Our findings provide an intuitive and objective reference for nephrologists to determine the optimal timing for hemodialysis preparation, including vascular access creation. This practical tool may aid in patient counseling, improve adherence to timely hemodialysis planning, and ultimately enhance patient outcomes by facilitating individualized ESKD life planning.

## Figures and Tables

**Table 1:** General information of patients at the time of hemodialysis initiation.

Characteristics	Values (n = 507)
Sex, male	299 (59.0)
Age (years)	61.0 ± 14.2
Body mass index (kg/m^2^)	24.6 ± 4.4
Physical activity	
Independent mobility	320 (63.1)
Requiring partial assistance	123 (24.3)
Requiring absolute assistance	64 (12.6)
Mean arterial pressure (mmHg)	101.8 ± 15.5
Serum creatinine (mg/dL)	9.2 ± 3.7
eGFR (mL/min/1.73 m^2^)	6.2 ± 2.8
Proteinuria (g/g)	3.3 ± 2.7
Left ventricular ejection fraction (%)	58.6 ± 10.5
Diabetes mellitus	296 (58.4)
Hypertension	485 (95.7)
Glomerulonephritis	49 (9.7)
Polycystic kidney disease	30 (5.9)
Cardiovascular disease	164 (32.3)
Stroke	82 (16.2)
Dementia	23 (4.5)
Malignancy	56 (11.0)
Liver cirrhosis	26 (5.1)
Medications	
RAAS inhibitor	343 (67.7)
Calcium channel blocker	389 (76.7)
Beta blocker	227 (44.8)
Diuretics	238 (46.9)

Data are shown as mean ± standard deviation for continuous variables or n (%) for categorical variables. eGFR, estimated glomerular filtration rate; RAAS, renin–angiotensin–aldosterone system.

**Table 2:** Mean eGFR levels according to the time before hemodialysis initiation.

Time Before Dialysis Initiation	eGFR (mL/min/1.73 m^2^)
Baseline (at the time of dialysis)	6.2 ± 2.8
1 month	8.2 ± 4.2
3 months	9.6 ± 4.8
6 months	11.7 ± 4.9
1 year	15.6 ± 7.0
2 years	22.9 ± 12.9
3 years	28.1 ± 15.2
4 years	33.0 ± 18.2
5 years	36.4 ± 19.3

Data are shown as mean ± standard deviation. eGFR, estimated glomerular filtration rate.

**Table 3:** Univariate analysis of predictors for eGFR 6 months before hemodialysis initiation.

Variables	β (95% CI)	*p*-Value
Sex, male	1.155 (0.285, 2.025)	0.009 *
Age	0.051 (0.021, 0.0808)	0.001 *
Body mass index	−0.003 (−0.102, 0.095)	0.947
Impaired mobility	2.311 (0.998, 3.625)	0.001 *
Diabetes mellitus	2.521 (1.675, 3.367)	<0.001 *
Duration of diabetes mellitus	0.042 (−0.023, 0.107)	0.205
Poorly controlled diabetes mellitus	1.615 (0.539, 2.691)	0.003 *
Hypertension	0.166 (−1.948, 2.280)	0.877
Duration of hypertension	0.020 (−0.031, 0.070)	0.440
Smoking	0.264 (−0.738, 1.266)	0.605
Cardiovascular disease	2.470 (1.575, 3.365)	<0.001 *
Stroke	2.000 (0.843, 3.157)	0.001 *
Dementia	3.895 (1.853, 5.937)	<0.001 *
Malignancy	0.789 (−0.583, 2.162)	0.259
Liver cirrhosis	2.792 (0.854, 4.729)	0.005 *
Echocardiographic findings		
Left ventricular ejection fraction	−0.111 (−0.159, −0.063)	<0.001 *
E/e’	0.109 (0.035, 0.183)	0.004 *
Regional wall motion abnormality	3.770 (2.230, 5.309)	<0.001 *
Medications		
RAAS inhibitor	−0.129 (−1.052, 0.795)	0.784
Calcium channel blocker	−0.531 (−1.549, 0.487)	0.306
Beta blocker	0.368 (−0.497, 1.234)	0.404
Statin	0.668 (−0.196, 1.531)	0.129
Nephrotic-range proteinuria	1.712 (0.818, 2.607)	<0.001 *
Hypoalbuminemia	2.495 (1.281, 3.708)	<0.001 *
BUN ≤ 60 mg/dL	4.756 (3.998, 5.513)	<0.001 *
Phosphorus ≤ 4.5 mg/dL	3.952 (3.151, 4.753)	<0.001 *
Volume overload	1.769 (0.920, 2.618)	<0.001 *

BUN, blood urea nitrogen; CI, confidence interval; E/e’, early diastolic mitral inflow velocity to early diastolic mitral annular velocity ratio; eGFR, estimated glomerular filtration rate; RAAS, renin–angiotensin–aldosterone system. * *p* < 0.05.

**Table 4:** Multivariate analysis of predictors for eGFR 6 months before hemodialysis initiation.

Variable	B (95% CI)	SE	β	*p*-Value
Intercept	11.394 (8.534, 14.253)	1.454		<0.001 *
Sex, male	1.129 (0.285, 1.972)	0.429	0.109	0.009 *
Impaired mobility	1.341 (0.122, 2.560)	0.620	0.091	0.031 *
Diabetes mellitus	0.996 (0.128, 1.863)	0.441	0.096	0.025 *
Cardiovascular disease	1.047 (0.166, 1.929)	0.448	0.101	0.020 *
Ejection fraction	−0.068 (−0.109, −0.027)	0.021	−0.140	0.001 *
BUN ≤ 60 mg/dL	3.315 (2.405, 4.224)	0.463	0.324	<0.001 *
Phosphorus ≤ 4.5 mg/dL	2.717 (1.835, 3.598)	0.448	0.266	<0.001 *

BUN, blood urea nitrogen; CI, confidence interval; eGFR, estimated glomerular filtration rate; SE, standard error. * *p* < 0.05.

## Data Availability

The data presented in this study are available on request from the corresponding author.

## Supplementary Materials

The following supporting information can be downloaded at: https://www.mdpi.com/article/10.3390/biomedicines13122960/s1. Table S1. The mean eGFR 6 months before hemodialysis initiation according to the glycemic control status in patients with diabetes mellitus; Table S2. The mean eGFR 6 months before hemodialysis initiation in patients without diabetes mellitus; Table S3. Characteristics of the development cohort and the internal validation cohort; Table S4. Characteristics of the development cohort and the internal and external validation cohort; Figure S1. Web-based calculator for predicting eGFR 6 months prior to hemodialysis initiation; Figure S2. Annual comparison of eGFR 6 months before and at initiation of hemodialysis; Figure S3. The trajectory of estimated glomerular filtration rate (eGFR) decline according to the glycemic control status in patients with diabetes mellitus (DM) (n = 296); Figure S4. The trajectory of estimated glomerular filtration rate (eGFR) decline according to the proteinuria severity in patients without diabetes mellitus (DM) (n = 201); Figure S5. Diagnostic plots of regression standardized residuals.

## Abbreviations

The following abbreviations are used in this manuscript:

CKD	Chronic kidney disease
ESKD	End-stage kidney disease
DM	Diabetes mellitus
eGFR	Estimated glomerular filtration rate
BUN	Blood urea nitrogen
PCKD	Polycystic kidney disease
LC	Liver cirrhosis
CVD	Cardiovascular disease
KDOQI	Kidney Disease Outcomes Quality Initiative
KRT	Kidney replacement therapy
EF	Ejection fraction
MAE	mean absolute error
RMSE	Root mean squared error
P30	Percentage of predicted values within 30% of the observed values,
