# Supplementary material for: Prediction Model for eGFR Thresholds Guiding the Optimal Timing of Hemodialysis Preparation in Chronic Kidney Disease
Source: Biomedicines. 2025 Dec 1;13(12):2960. doi: 10.3390/biomedicines13122960 (PMC12730943; doi:10.3390/biomedicines13122960)
Supplement: Supplementary file 1 [file biomedicines-13-02960-s001.zip › biomedicines-3918588-supplementary.pdf]

Supplementary Materials

Supplementary Table S1. The mean eGFR 6 months before hemodialysis initiation according to the glycemic control status in patients with diabetes mellitus.

Variable	Overall	Poorly-Controlled DM	Well-Controlled DM	*p*-Value
eGFR_6M (mL/min/1.73 m^2)	12.7 ± 5.0	13.3 ± 4.9	12.2 ± 4.9	0.003 *

Data are shown as mean ± standard deviation. DM, diabetes mellitus; eGFR, estimated glomerular filtration rate; eGFR_6M, eGFR 6 months before hemodialysis initiation. * $p < 0.05$.

Supplementary Table S2. The mean eGFR 6 months before hemodialysis initiation in patients without diabetes mellitus.

Variable	eGFR_6M (mL/min/1.73 m²)		*p*-Value
	Disease	Control	
Overall	10.2 ± 4.5	N/A	N/A
Cardiovascular disease	12.5 ± 6.1	9.4 ± 3.5	<0.001 *
Stroke	12.4 ± 5.3	9.9 ± 4.4	0.012 *
Nephtoric-range proteinuria	13.0 ± 4.5	9.9 ± 4.3	0.002 *

Data are shown as mean ± standard deviation. DM, Diabetes mellitus; eGFR, estimated glomerular filtration rate; eGFR_6M, eGFR 6 months before hemodialysis initiation; N/A, Not applicable. * $p < 0.05$.

Supplementary Table S3. Characteristics of the development cohort and the internal validation cohort.

Characteristics	Development Cohort (n = 507)	Internal Validation Cohort (n = 50)	*p*-Value
Sex, male	299 (59.0)	28 (56.0)	0.684
Impaired mobility	64 (12.6)	7 (14.0)	0.785
Diabetes mellitus	296 (58.4)	29 (58.0)	0.958
Cardiovascular disease	164 (32.3)	7 (14.0)	0.007 *
Left ventricular ejection fraction (%)	58.6 ± 10.5	60.9 ± 8.0	0.139
BUN ≤ 60 mg/dL	273 (53.8)	22 (44.0)	0.184
Phosphorus ≤ 4.5 mg/dL	209 (41.2)	18 (36.0)	0.421
eGFR_6M (mL/min/1.73 m^2)	11.7 ± 4.9	10.9 ± 3.4	0.166

Data are shown as mean ± standard deviation for continuous variables or n (%) for categorical variables. BUN, blood urea nitrogen; eGFR_6M, eGFR 6 months before hemodialysis initiation. * $p < 0.05$.

Supplementary Table S4. Characteristics of the development cohort and the internal and external validation cohort.

Characteristics	Development Cohort (n = 507)	Internal/External Validation Cohort (n = 181)	*p*-Value
Sex, male	299 (59.0)	93 (51.4)	0.077
Age	61.0 ± 14.2	61.2 ± 14.1	0.675
Impaired mobility	64 (12.6)	19 (10.5)	0.446
Diabetes mellitus	296 (58.4)	111 (61.3)	0.489
Cardiovascular disease	164 (32.3)	35 (19.3)	<0.001
Left ventricular ejection fraction (%)	58.6 ± 10.5	61.5 ± 9.1	0.320
BUN ≤ 60 mg/dL	273 (53.8)	106 (58.6)	0.273
Phosphorus ≤ 4.5 mg/dL	209 (41.2)	95 (52.5)	0.014
eGFR_6M (mL/min/1.73 m^2)	11.7 ± 4.9	13.3 ± 5.3	0.364

Data are shown as mean ± standard deviation for continuous variables or n (%) for categorical variables. BUN, blood urea nitrogen; eGFR_6M, eGFR 6 months before hemodialysis initiation. * $p < 0.05$.

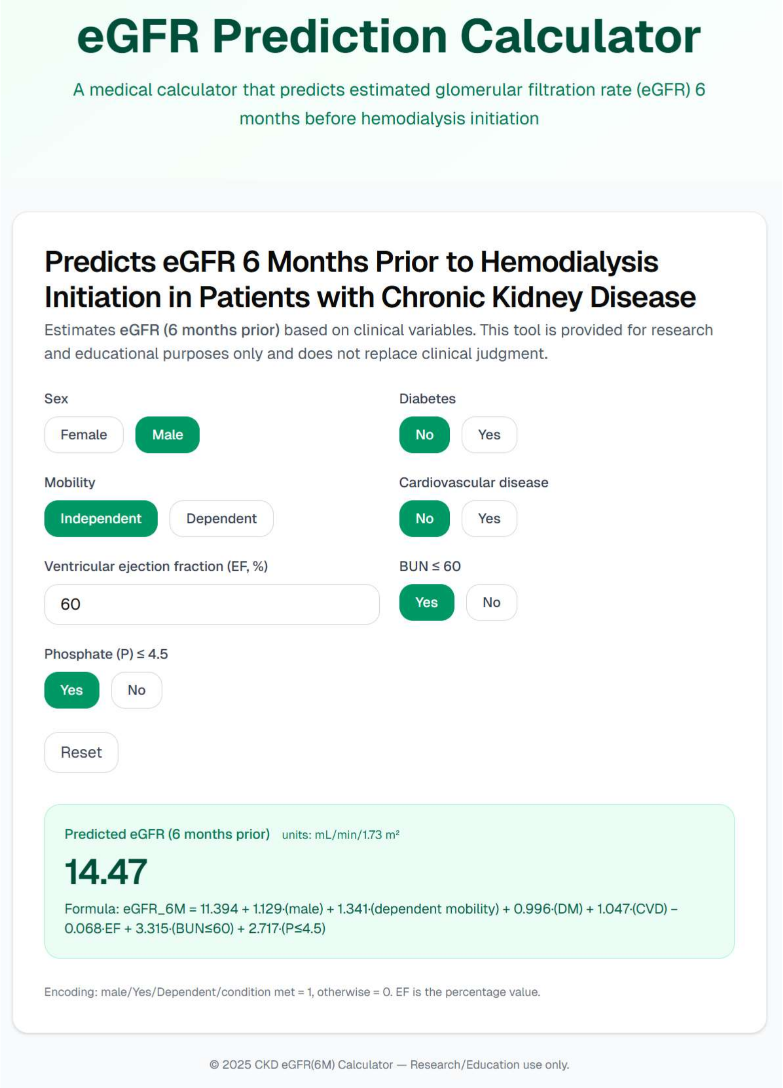

Supplementary Figure S1. Web-based calculator for predicting eGFR 6 months prior to hemodialysis initiation.

Screenshot of the online calculator (https://v0-e-gfr-calculator.vercel.app/) developed from the study's prediction equation to estimate the glomerular filtration rate 6 months before hemodialysis initiation (eGFR_6M) in patients with chronic kidney disease. The tool incorporates sex, diabetes status, mobility, cardiovascular disease, ventricular ejection fraction, serum blood urea nitrogen and phosphate levels into a regression formula and displays the predicted eGFR_6M (mL/min/1.73 m^2) together with the underlying equation.

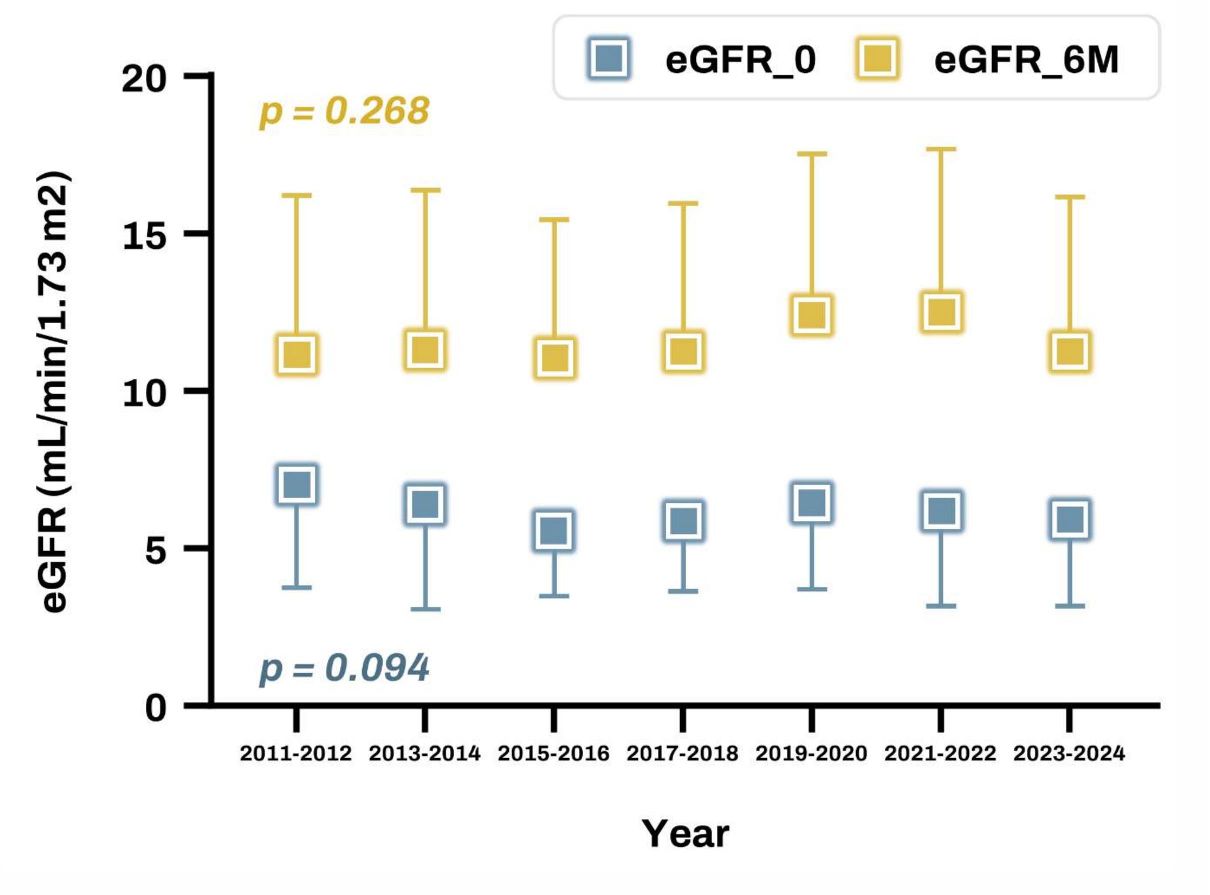

Supplementary Figure S2. Annual comparison of eGFR 6 months before and at initiation of hemodialysis.

Mean eGFR values 6 months before hemodialysis initiation (eGFR_6M) and at the time of hemodialysis initiation (eGFR_0) are shown according to calendar period of hemodialysis initiation. Data are presented as mean ± SD. P values were obtained from one-way ANOVA comparing mean eGFR values across calendar period.

eGFR, estimated glomerular filtration rate; eGFR_6M, eGFR 6 months prior to hemodialysis initiation; eGFR_0, eGFR at the time of hemodialysis initiation.

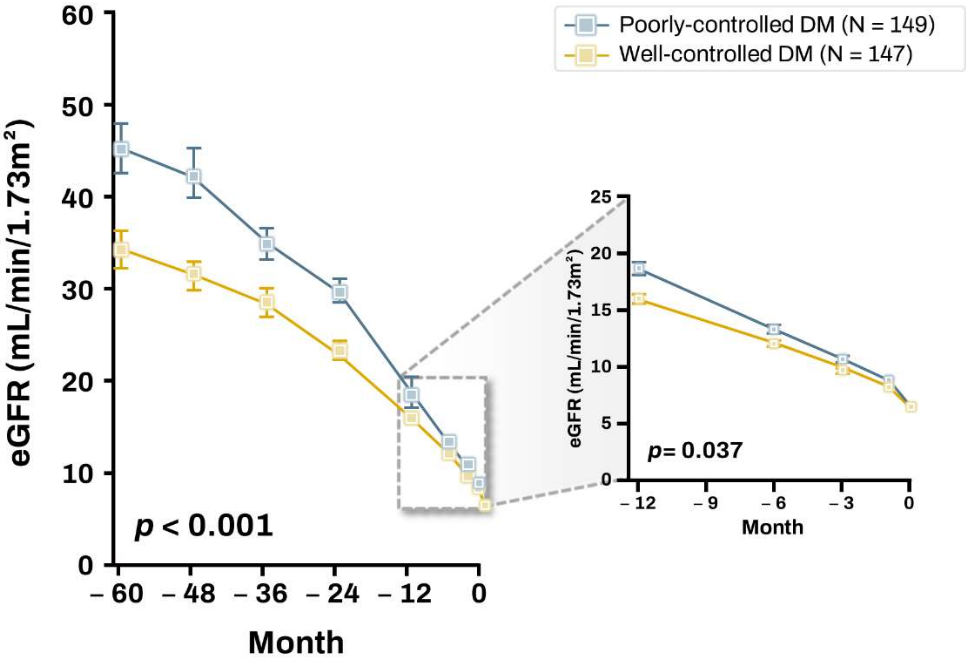

Supplementary Figure S3. The trajectory of estimated glomerular filtration rate (eGFR) decline according to the glycemic control status in patients with diabetes mellitus (DM) (n = 296). Data are shown as mean ± standard error.

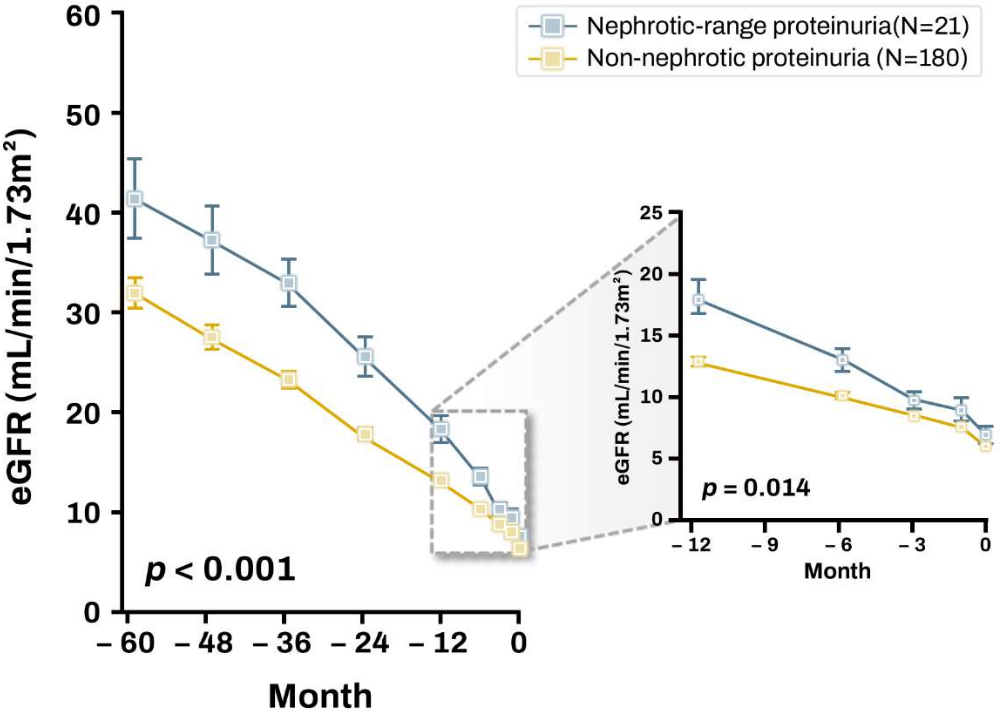

Supplementary Figure S4. The trajectory of estimated glomerular filtration rate (eGFR) decline according to the proteinuria severity in patients without diabetes mellitus (DM) (n = 201). Data are shown as mean ± standard error.

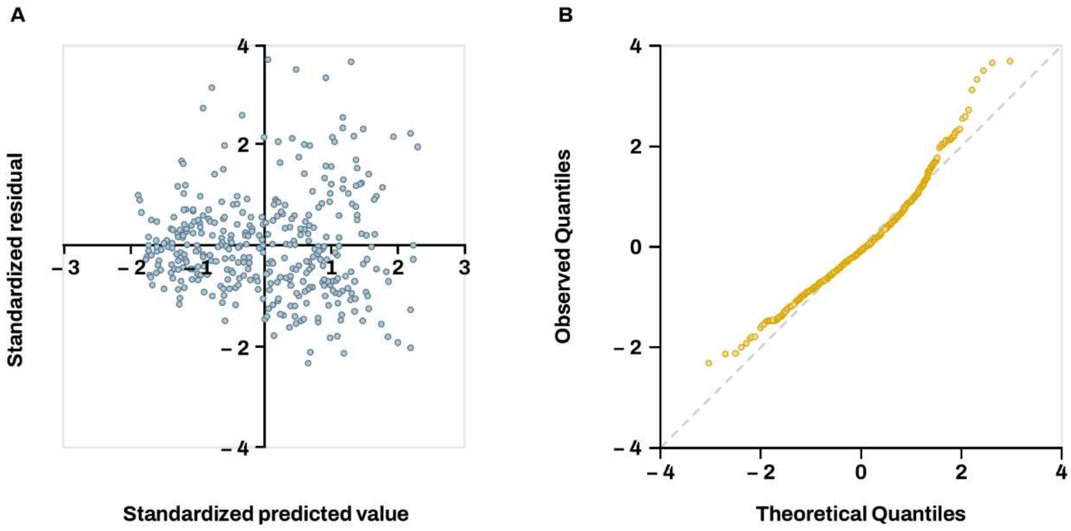

Supplementary Figure S5. Diagnostic plots of regression standardized residuals. (**A**) Scatter plot of residuals versus fitted values. (**B**) Normal quantile-quantile plot of residuals.
